# Author Correction: Statistical determinants of visuomotor adaptation along different dimensions during naturalistic 3D reaches

**DOI:** 10.1038/s41598-022-16148-9

**Published:** 2022-07-07

**Authors:** E. Ferrea, J. Franke, P. Morel, A. Gail

**Affiliations:** 1 Cognitive Neuroscience Laboratory, German Primate Center, Leibniz Institute for Primate Research, Göttingen, Germany; 2 Georg-Elias-Mueller Institute of Psychology, University of Goettingen, Göttingen, Germany; 3 Bernstein Center for Computational Neuroscience, Göttingen, Germany; 4 Univ. Littoral Côte d’Opale, Univ. Artois, Univ. Lille, ULR 7369 - URePSSS - Unité de Recherche Pluridisciplinaire Sport Santé Sociéte, F-62100 Calais, France

Correction to: *Scientific Reports* 10.1038/s41598-022-13866-y, published online 17 June 2022

The original version of this Article contained an error in Affiliation 4, which was incorrectly given as ‘ULR 7369 - URePSSS - Unite de Recherche Pluridisciplinaire Sport Santé Sociéte, Univ. Littoral Côte d’Opale, Univ. Lille, Univ. Artois, Calais, France’.

The correct affiliation is listed below.

Univ. Littoral Côte d’Opale, Univ. Artois, Univ. Lille, ULR 7369 - URePSSS - Unité de Recherche Pluridisciplinaire Sport Santé Société, F-62100 Calais, France

The original Article has been corrected.

