# Supplementary material for: Statistical determinants of visuomotor adaptation along different dimensions during naturalistic 3D reaches
Source: Sci Rep. 2022 Jun 17;12:10198. doi: 10.1038/s41598-022-13866-y (PMC9205902; doi:10.1038/s41598-022-13866-y)
Supplement: Supplementary file 1 — Supplementary Legends. [file 41598_2022_13866_MOESM1_ESM.docx]

**Supplementary Information**

Supplementary Figure 1. Results of fitting the hierarchical HMC model to the adaptation profile of each subject (n = 24) with perturbation applied to the Sagittal plane.

Supplementary Figure 2. Results of fitting the hierarchical HMC model to the adaptation profile of each subject (n = 24) with perturbation applied to the horizontal plane.

Supplementary Figure 3. Results of fitting the hierarchical HMC model to the adaptation profile of each subject (n = 24) with perturbation applied to the coronal plane.

Supplementary Figure 4. Similar to figure 5 but with the model applied on a trial base rather than epoch base (no average over 8 consecutive trials). A) Example of single subject time course of adaptation with 2-state model fits. B) Extrapolated model parameter for all the subjects (different colors). C) Slow learning rate (bs) vs Kalman gain relationship.
