# Supplementary figures and images for: Statistical determinants of visuomotor adaptation along different dimensions during naturalistic 3D reaches
Source: Sci Rep. 2022 Jun 17;12:10198. doi: 10.1038/s41598-022-13866-y (PMC9205902; doi:10.1038/s41598-022-13866-y)

# All Subjects Sagittal perturbation

• data      — Slow state      — Fast state      — Fast State + Slow state

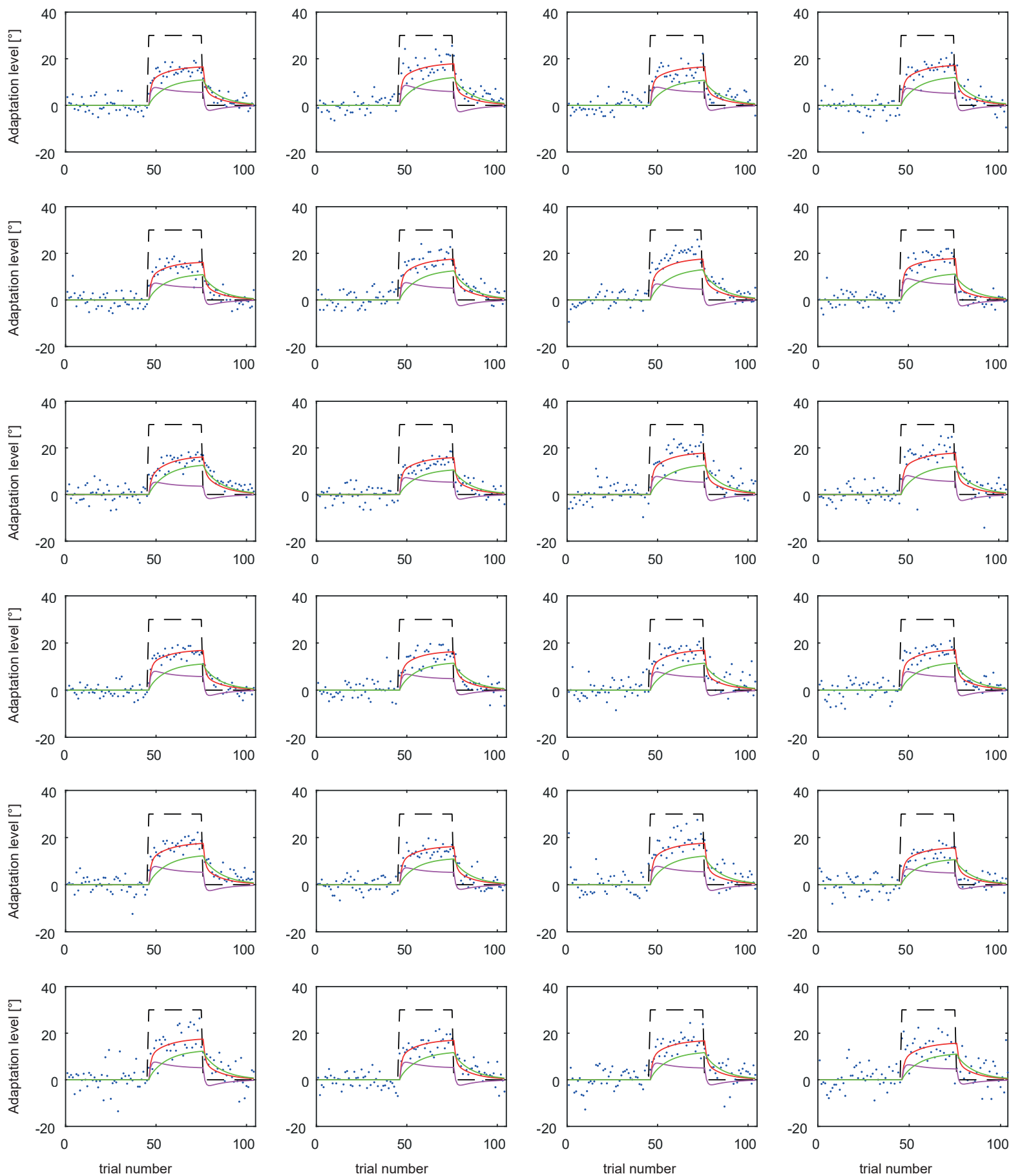

Supplement: Supplementary file 2 — Supplementary Figure 1. [file 41598_2022_13866_MOESM2_ESM.pdf]

# All Subjects Horizontal perturbation

• data      — Slow state      — Fast state      — Fast State + Slow state

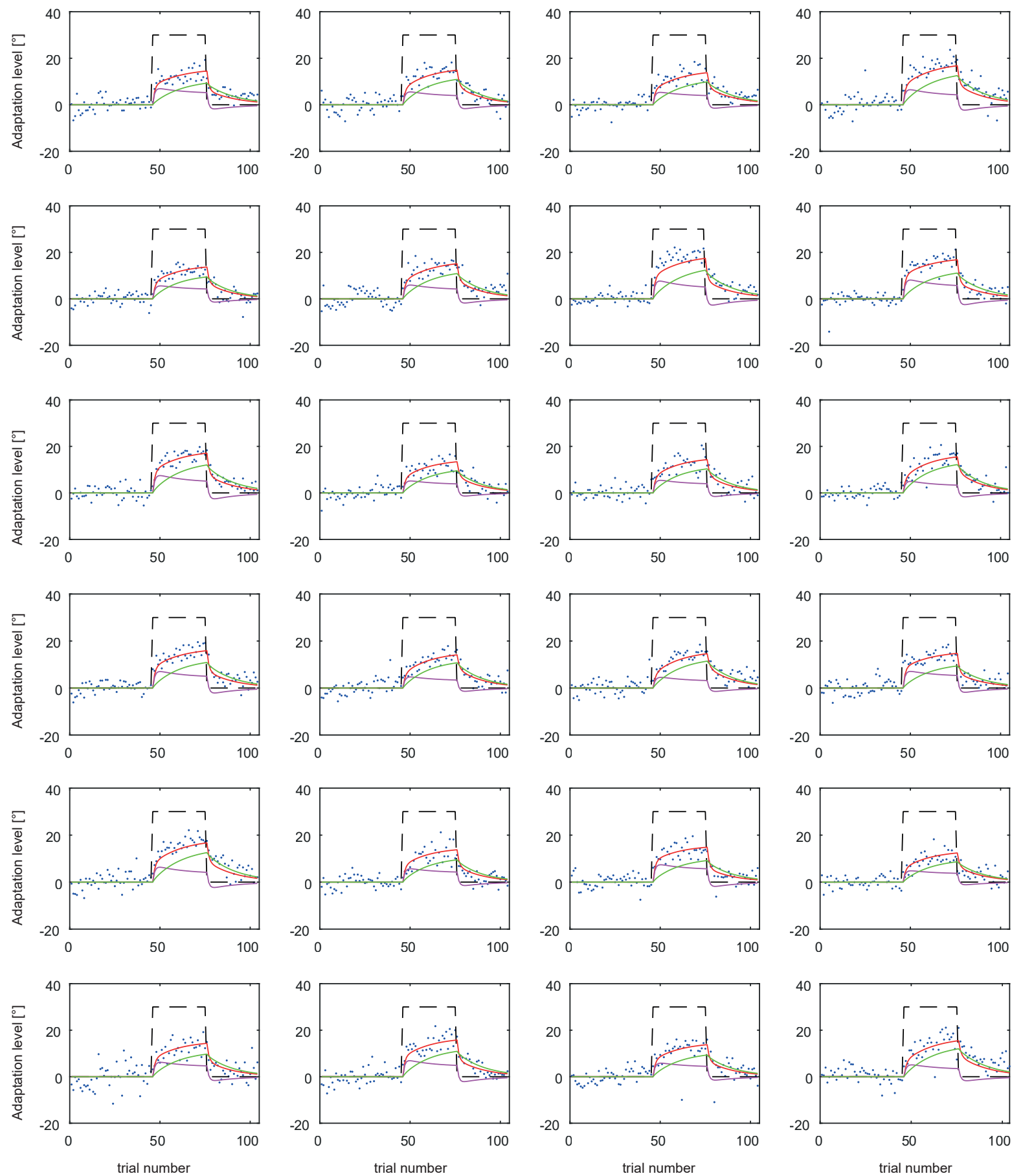

Supplement: Supplementary file 3 — Supplementary Figure 2. [file 41598_2022_13866_MOESM3_ESM.pdf]

# All Subjects Coronal perturbation

• data      — Slow state      — Fast state      — Fast State + Slow state

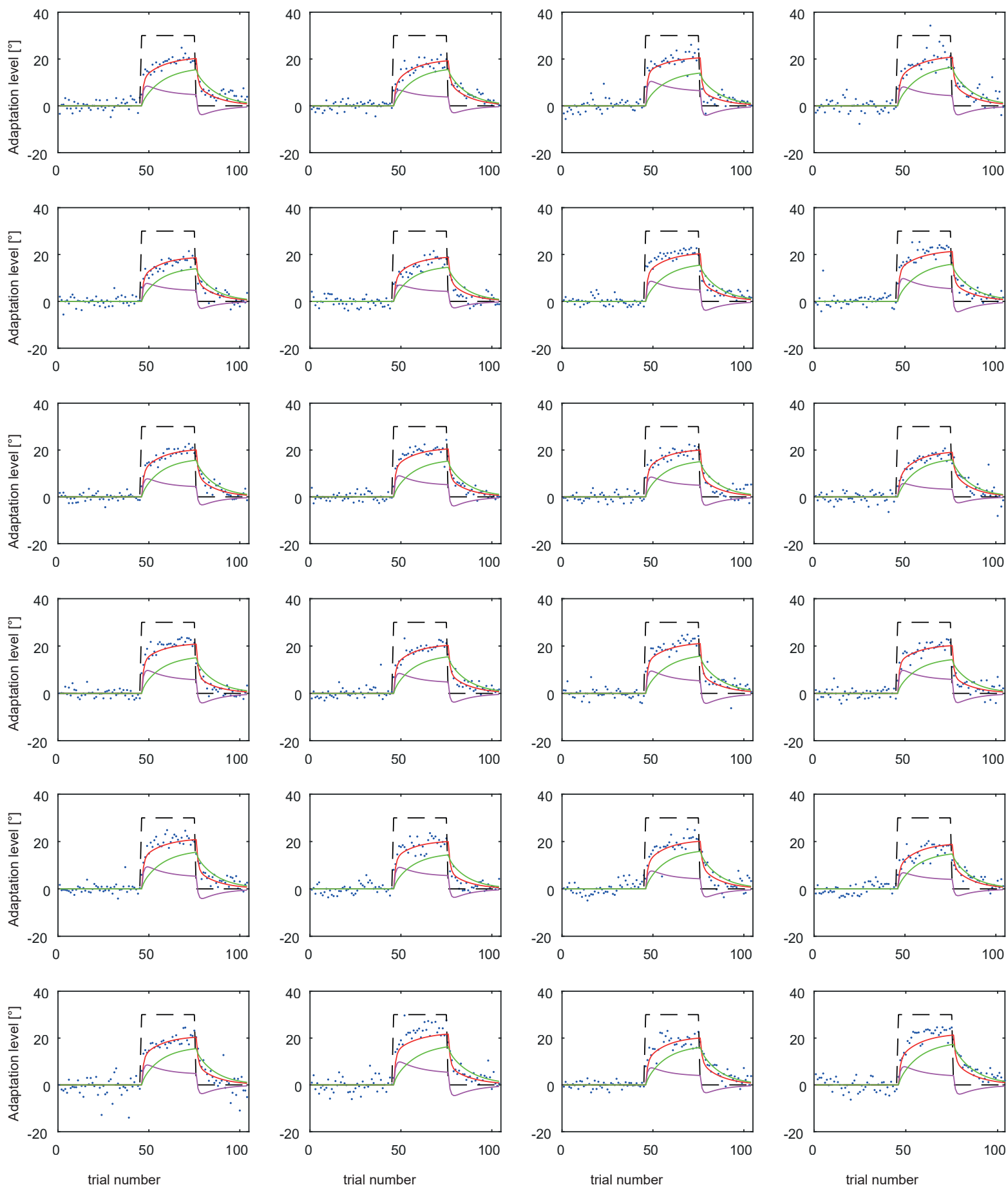

Supplement: Supplementary file 4 — Supplementary Figure 3. [file 41598_2022_13866_MOESM4_ESM.pdf]

A

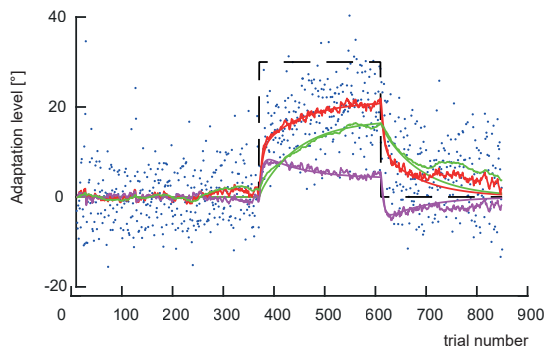

B

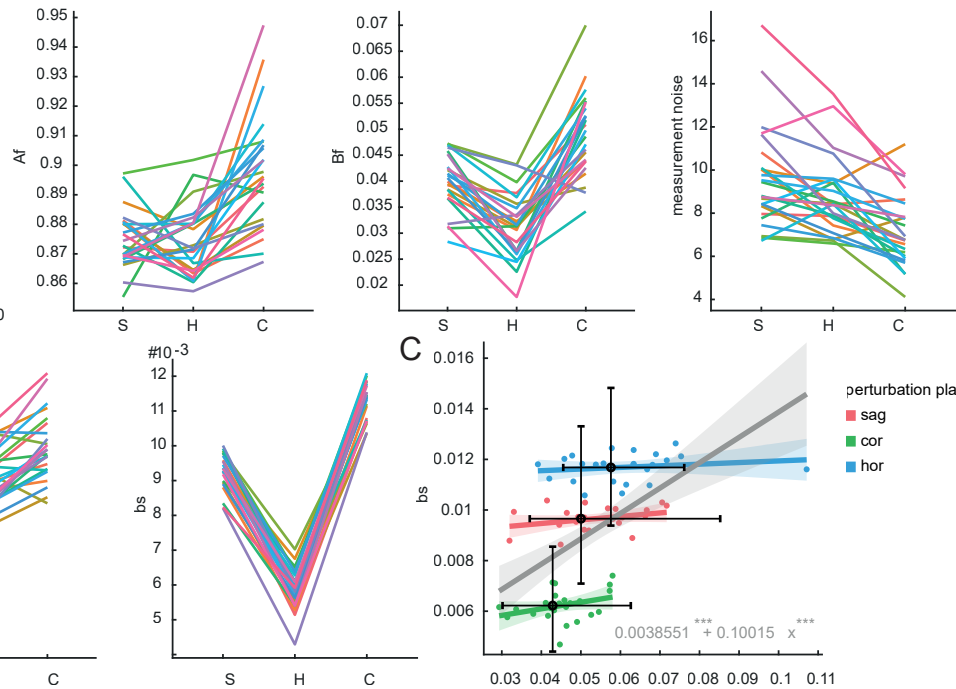

C

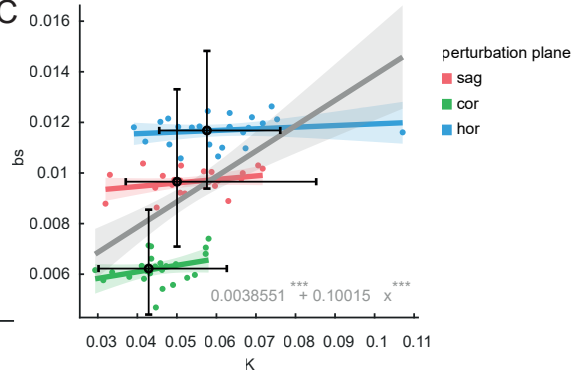

Supplement: Supplementary file 5 — Supplementary Figure 4. [file 41598_2022_13866_MOESM5_ESM.pdf]
